# (*S*)-2-[(2*R*,3*S*)-2-Ammonio-3-hydr­oxy-3-(4-nitro­phen­yl)propanamido]-4-methyl­penta­noate monohydrate

**DOI:** 10.1107/S1600536808039056

**Published:** 2008-11-26

**Authors:** Kang-Hui Yang, Xun Li, Jian-Zhi Gong, Hao Fang, Wen-Fang Xu

**Affiliations:** a Institute of Medicinal Chemistry, School of Pharmacy, Shandong University, Ji’nan 250012, People’s Republic of China

## Abstract

The structure of the title compound, C_15_H_21_N_3_O_6_·H_2_O, is of inter­est with respect to assumed anti­cancer activity. The title mol­ecules are linked through inter­molecular O—H⋯O hydrogen-bonded chains along the *a* axis. These chains are connected by inter­molecular N—H⋯O hydrogen bonds through the crystallographic screw axis along [010], forming layers, which are stabilized by other N—H⋯O bonds with water O atoms as acceptors and O—H⋯O bonds with water H atoms as donors. The H atoms of the protonated amino cation are also involved in inter­molecular N—H⋯O bonding inter­actions.

## Related literature

For various medicinal agents similar to the title compound, see: Shinagawa *et al.* (1987 ▶); Shin & Pyo (1984 ▶). For anti-cancer and anti-inflammatory biological properties, see: Aozuka *et al.* (2004 ▶). For amino­peptidase N (APN/CD13) inhibitors, see: Xu & Li (2005 ▶). For the synthesis of the starting material, see: Testa *et al.* (2004 ▶).
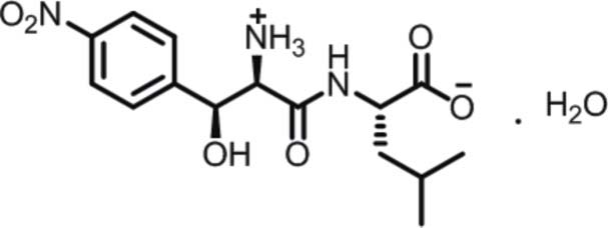


## Experimental

### 

#### Crystal data

C_15_H_21_N_3_O_6_·H_2_O
*M* *_r_* = 357.36
Monoclinic, 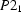

*a* = 8.9787 (7) Å
*b* = 6.7850 (5) Å
*c* = 14.7148 (11) Å
β = 95.362 (5)°
*V* = 892.51 (12) Å^3^
*Z* = 2
Mo *K*α radiation
μ = 0.11 mm^−1^
*T* = 296 (2) K
0.50 × 0.20 × 0.15 mm

#### Data collection

Bruker APEXII CCD area-detector diffractometer
Absorption correction: multi-scan (*SADABS*; Bruker, 2005 ▶) *T*_min_ = 0.949, *T*_max_ = 0.984
8245 measured reflections
2254 independent reflections
1853 reflections with *I* > 2σ(*I*)
*R*_int_ = 0.028

#### Refinement

*R*[*F* ^2^ > 2σ(*F* ^2^)] = 0.041
*wR*(*F* ^2^) = 0.104
*S* = 1.04
2254 reflections
243 parameters
1 restraint
H atoms treated by a mixture of independent and constrained refinement
Δρ_max_ = 0.19 e Å^−3^
Δρ_min_ = −0.16 e Å^−3^

### 

Data collection: *APEX2* (Bruker, 2005 ▶); cell refinement: *APEX2* and *SAINT* (Bruker, 2005 ▶); data reduction: *SAINT*; program(s) used to solve structure: *SHELXS97* (Sheldrick, 2008 ▶); program(s) used to refine structure: *SHELXL97* (Sheldrick, 2008 ▶); molecular graphics: *SHELXTL* (Sheldrick, 2008 ▶); software used to prepare material for publication: *WinGX* (Farrugia, 1999 ▶).

## Supplementary Material

Crystal structure: contains datablocks I, global. DOI: 10.1107/S1600536808039056/si2128sup1.cif

Structure factors: contains datablocks I. DOI: 10.1107/S1600536808039056/si2128Isup2.hkl

Additional supplementary materials:  crystallographic information; 3D view; checkCIF report

## Figures and Tables

**Table 1:** Hydrogen-bond geometry (Å, °)

*D*—H⋯*A*	*D*—H	H⋯*A*	*D*⋯*A*	*D*—H⋯*A*
O7—H7*W*⋯O5	0.92	1.87	2.679 (2)	146
O7—H8*W*⋯O4^i^	0.88	1.96	2.734 (3)	146
N2—H2*B*⋯O3^ii^	0.87 (3)	1.99 (3)	2.854 (3)	172 (3)
O3—H4⋯O5^iii^	0.82	1.79	2.612 (2)	175
N3—H3*A*⋯O7^i^	0.86	2.08	2.914 (3)	163
N2—H2*C*⋯O6^i^	0.99 (3)	1.96 (3)	2.811 (3)	143 (3)
N2—H2*A*⋯O7	0.95 (3)	1.89 (3)	2.801 (2)	158 (3)

Symmetry codes: (i) 
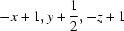
; (ii) 
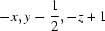
; (iii) 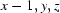.

**Fig. 1.**
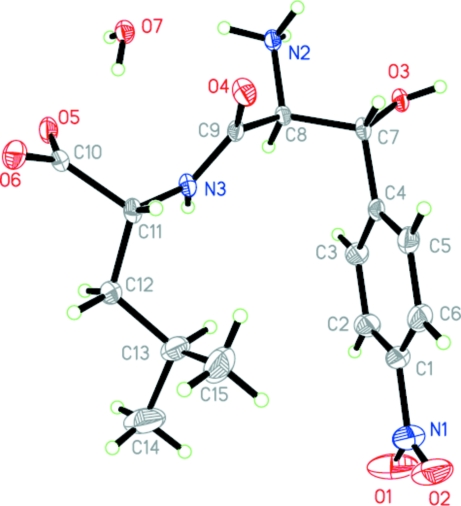
A view of the title molecules, showing 30% probability displacement ellipsoids and the numbering scheme of non-hydrogen atoms.

**Fig. 2.**
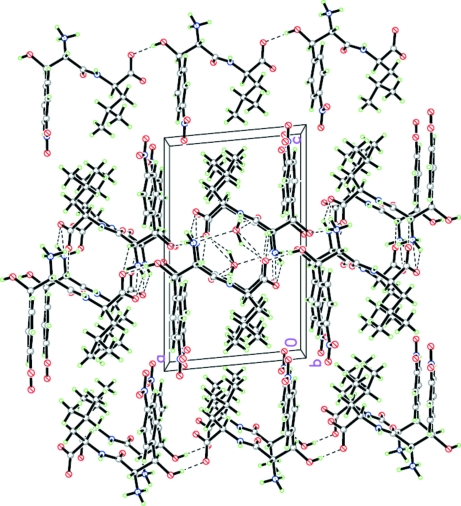
A view of the unit cell of the title compound, showing intermolecular hydrogen bonds as dashed lines.

## supplementary crystallographic information

### Comment

The 2-amino-1-hydroxy carboxylic acids and 1-amino-2-hydroxy carboxylic acids are important precursor and scaffold of various medicinal agents (Shinagawa *et al.*, 1987). It was reported that many of their derivatives exhibit anti-cancer and anti-inflammatory properties (Aozuka *et al.*, 2004). Our lab has been engaged in developing 1-amino-2-hydroxy carboxylic acids derivatives as Aminopeptidase N (APN/CD13) inhibitors (Xu & Li, 2005). One of the aims of the projects is to use different amino acid coupled with 1-amino-2-hydroxy carboxylic acids scaffold, the resulting target derivatives are hope to have antitumor activities. Both the title compound (Fig. 1) and the reported chloramphenicol base have the amino-hydroxy-nitrophenyl scaffold in their structures (Shin & Pyo, 1984). In the molecule, the bond length of C8–N2 is 1.489 (3) Å, which is slightly longer than that of the C8–N2 (1.473 (7) Å) in the chloramphenicol base. This is probably due to the electrostatic attractions between the carboxyl anion and the protonated amino cation. The title molecules are linked through intermolecular O—H···O hydrogen bonded chains along the *a* axis (Table 1, Fig. 2). These chains are connected by intermolecular N—H···O hydrogen bonds through the crystallographic screw axis along the [0 1 0] direction to form layers which are stabilized by other N—H···O bonds with water oxygen atoms as acceptors and O—H···O bonds with water hydrogen atoms as donors.The H atoms of the protonated amino cation were also involved in intermolecular N—H···O bonding interactions.

### Experimental

The starting material, (2*R*,3*S*)-2-(*tert*-butoxycarbonylamino) -3-hydroxy-3-(4-nitrophenyl) propanoic acid, prepared according to the literature (Testa *et al.*, 2004), was coupled with *L*-leucine methyl ester by using dicyclohexylcarbodiimide (DCC) and 1-hydroxybenzotriazole (HOBt). Finally, the title compound was yielded by hydrolyzing in NaOH and then deprotecting Boc group with hydrochloride. Crystals appropriate for data collection were obtained by slow evaporation of the solid in methanol at room temperature. Yield 40%, m.p. 481 K.

### Refinement

All H atoms were positioned geometrically using a riding model with C—H = 0.92–1.02 Å, N—H = 0.86 and O—H = 0.82 Å. Their isotropic displacement parameters were set to 1.2 times (1.5 times for CH_3_ groups) the equivalent displacement parameter of their parent atoms. In addition, the three H atoms at N2 were located from a difference Fourier map and refined isotropically, with N–H distances in the range 0.88 (3)–0.92 (3) Å with *U*_iso_(H) = 1.3–1.75 times *U*_eq_(N2).

In the absence of significant anomalous dispersion effects, 1625 Friedel pairs were averaged.

### Crystal data

**Table:**

C_15_H_21_N_3_O_6_·H_2_O	*F*_000_ = 380
*M**_r_* = 357.36	*D*_x_ = 1.330 Mg m^−^^3^
Monoclinic, *P*2_1_	Melting point = 556.2–558.6 K
Hall symbol: P 2yb	Mo *K*α radiation λ = 0.71073 Å
*a* = 8.9787 (7) Å	Cell parameters from 2639 reflections
*b* = 6.7850 (5) Å	θ = 2.8–24.2º
*c* = 14.7148 (11) Å	µ = 0.11 mm^−^^1^
β = 95.362 (5)º	*T* = 296 (2) K
*V* = 892.51 (12) Å^3^	Prism, colourless
*Z* = 2	0.50 × 0.20 × 0.15 mm

### Data collection

**Table:**

Bruker APEXII CCD area-detector diffractometer	2254 independent reflections
Radiation source: fine-focus sealed tube	1853 reflections with *I* > 2σ(*I*)
Monochromator: graphite	*R*_int_ = 0.028
*T* = 296(2) K	θ_max_ = 27.6º
φ and ω scans	θ_min_ = 2.3º
Absorption correction: multi-scan(SADABS; Bruker, 2005)	*h* = −11→11
*T*_min_ = 0.949, *T*_max_ = 0.984	*k* = −8→8
8245 measured reflections	*l* = −19→18

### Refinement

**Table:**

Refinement on *F*^2^	Secondary atom site location: difference Fourier map
Least-squares matrix: full	Hydrogen site location: inferred from neighbouring sites
*R*[*F*^2^ > 2σ(*F*^2^)] = 0.041	H atoms treated by a mixture of independent and constrained refinement
*wR*(*F*^2^) = 0.105	*w* = 1/[σ^2^(*F*_o_^2^) + (0.059*P*)^2^ + 0.0778*P*] where *P* = (*F*_o_^2^ + 2*F*_c_^2^)/3
*S* = 1.04	(Δ/σ)_max_ < 0.001
2254 reflections	Δρ_max_ = 0.19 e Å^−^^3^
243 parameters	Δρ_min_ = −0.16 e Å^−^^3^
1 restraint	Extinction correction: none
Primary atom site location: structure-invariant direct methods	

### Special details

**Table:**

Geometry. All e.s.d.'s (except the e.s.d. in the dihedral angle between two l.s. planes) are estimated using the full covariance matrix. The cell e.s.d.'s are taken into account individually in the estimation of e.s.d.'s in distances, angles and torsion angles; correlations between e.s.d.'s in cell parameters are only used when they are defined by crystal symmetry. An approximate (isotropic) treatment of cell e.s.d.'s is used for estimating e.s.d.'s involving l.s. planes.
Refinement. Refinement of *F*^2^ against ALL reflections. The weighted *R*-factor *wR* and goodness of fit *S* are based on *F*^2^, conventional *R*-factors *R* are based on *F*, with *F* set to zero for negative *F*^2^. The threshold expression of *F*^2^ > σ(*F*^2^) is used only for calculating *R*-factors(gt) *etc*. and is not relevant to the choice of reflections for refinement. *R*-factors based on *F*^2^ are statistically about twice as large as those based on *F*, and *R*- factors based on ALL data will be even larger.

### Fractional atomic coordinates and isotropic or equivalent isotropic displacement parameters (Å^2^)

**Table:**

	*x*	*y*	*z*	*U*_iso_*/*U*_eq_	
C1	0.1171 (3)	1.0193 (5)	0.8450 (2)	0.0593 (8)	
C2	0.1386 (3)	1.1114 (5)	0.7646 (2)	0.0576 (8)	
H2	0.1620	1.2449	0.7637	0.069*	
C3	0.1250 (3)	1.0037 (4)	0.68503 (19)	0.0466 (6)	
H10	0.1388	1.0647	0.6298	0.056*	
C4	0.0906 (2)	0.8025 (4)	0.68693 (16)	0.0350 (5)	
C5	0.0699 (3)	0.7141 (5)	0.76921 (18)	0.0487 (7)	
H5	0.0466	0.5806	0.7708	0.058*	
C6	0.0834 (3)	0.8219 (6)	0.8498 (2)	0.0627 (9)	
H6	0.0700	0.7624	0.9053	0.075*	
C7	0.0792 (2)	0.6835 (4)	0.59945 (16)	0.0320 (5)	
H7	0.0586	0.5457	0.6137	0.038*	
C8	0.2268 (2)	0.6944 (3)	0.55531 (14)	0.0303 (5)	
H8	0.2570	0.8326	0.5509	0.036*	
C9	0.3482 (2)	0.5812 (4)	0.61333 (15)	0.0313 (5)	
C10	0.7060 (2)	0.5166 (4)	0.64655 (17)	0.0386 (6)	
C11	0.5895 (2)	0.6028 (4)	0.70569 (15)	0.0368 (5)	
H11	0.5533	0.4984	0.7441	0.044*	
C12	0.6643 (3)	0.7644 (5)	0.76667 (18)	0.0469 (6)	
H12A	0.7440	0.7047	0.8064	0.056*	
H12B	0.7100	0.8583	0.7280	0.056*	
C13	0.5625 (4)	0.8776 (7)	0.8260 (2)	0.0707 (10)	
H13	0.4790	0.9317	0.7860	0.085*	
C14	0.6501 (6)	1.0509 (8)	0.8721 (3)	0.1067 (16)	
H14A	0.7269	1.0011	0.9159	0.160*	
H14B	0.5832	1.1331	0.9025	0.160*	
H14C	0.6950	1.1266	0.8268	0.160*	
C15	0.4992 (5)	0.7477 (10)	0.8948 (3)	0.1045 (17)	
H15A	0.5792	0.6944	0.9353	0.157*	
H15B	0.4442	0.6420	0.8640	0.157*	
H15C	0.4337	0.8232	0.9292	0.157*	
N1	0.1333 (4)	1.1348 (8)	0.9301 (3)	0.0933 (13)	
N2	0.2080 (2)	0.6066 (3)	0.46217 (14)	0.0345 (4)	
N3	0.46309 (18)	0.6869 (3)	0.64893 (13)	0.0346 (4)	
H3A	0.4634	0.8115	0.6381	0.042*	
O1	0.1562 (6)	1.3125 (7)	0.9239 (3)	0.1463 (17)	
O2	0.1267 (4)	1.0520 (8)	1.0013 (2)	0.1297 (16)	
O3	−0.03561 (16)	0.7538 (3)	0.53411 (11)	0.0371 (4)	
H4	−0.1147	0.6988	0.5420	0.056*	
O4	0.33299 (19)	0.4031 (3)	0.62517 (14)	0.0482 (5)	
O5	0.71194 (18)	0.5906 (3)	0.56790 (12)	0.0471 (5)	
O6	0.7901 (2)	0.3852 (3)	0.67915 (14)	0.0548 (5)	
O7	0.50705 (17)	0.5833 (3)	0.42343 (12)	0.0410 (4)	
H7W	0.5421	0.5886	0.4840	0.086 (12)*	
H8W	0.5327	0.6792	0.3873	0.071 (10)*	
H2B	0.154 (3)	0.500 (5)	0.4578 (19)	0.045 (8)*	
H2C	0.162 (4)	0.702 (5)	0.416 (2)	0.055 (8)*	
H2A	0.305 (4)	0.564 (5)	0.448 (2)	0.060 (9)*	

### Atomic displacement parameters (Å^2^)

**Table:**

	*U*^11^	*U*^22^	*U*^33^	*U*^12^	*U*^13^	*U*^23^
C1	0.0459 (16)	0.076 (2)	0.0564 (18)	0.0056 (15)	0.0037 (13)	−0.0238 (17)
C2	0.0583 (18)	0.0473 (16)	0.0666 (19)	0.0060 (14)	0.0025 (14)	−0.0134 (16)
C3	0.0478 (15)	0.0390 (14)	0.0530 (16)	0.0044 (12)	0.0046 (12)	−0.0038 (13)
C4	0.0207 (10)	0.0405 (13)	0.0438 (13)	0.0056 (9)	0.0035 (9)	−0.0001 (11)
C5	0.0405 (14)	0.0568 (18)	0.0502 (15)	−0.0030 (12)	0.0117 (11)	0.0034 (14)
C6	0.0536 (17)	0.092 (3)	0.0436 (16)	−0.0035 (18)	0.0122 (13)	0.0009 (17)
C7	0.0198 (9)	0.0319 (10)	0.0443 (12)	0.0009 (9)	0.0030 (8)	0.0040 (10)
C8	0.0212 (10)	0.0278 (10)	0.0420 (12)	−0.0012 (8)	0.0041 (8)	−0.0011 (10)
C9	0.0234 (10)	0.0322 (12)	0.0389 (11)	0.0043 (9)	0.0063 (8)	−0.0008 (10)
C10	0.0236 (10)	0.0444 (14)	0.0469 (14)	−0.0012 (10)	−0.0007 (9)	−0.0097 (11)
C11	0.0246 (10)	0.0462 (14)	0.0394 (12)	0.0048 (10)	0.0019 (8)	0.0056 (11)
C12	0.0376 (13)	0.0602 (16)	0.0419 (13)	0.0076 (12)	−0.0014 (10)	−0.0077 (13)
C13	0.0663 (19)	0.099 (3)	0.0454 (16)	0.034 (2)	−0.0050 (14)	−0.0190 (18)
C14	0.143 (4)	0.097 (3)	0.079 (3)	0.024 (3)	−0.001 (3)	−0.040 (3)
C15	0.084 (3)	0.170 (5)	0.064 (2)	0.017 (3)	0.0297 (19)	−0.009 (3)
N1	0.085 (2)	0.126 (4)	0.069 (2)	0.004 (2)	0.0054 (17)	−0.041 (2)
N2	0.0247 (10)	0.0361 (11)	0.0433 (11)	−0.0023 (9)	0.0057 (8)	−0.0012 (10)
N3	0.0223 (9)	0.0356 (10)	0.0458 (11)	−0.0005 (8)	0.0027 (7)	0.0025 (9)
O1	0.222 (5)	0.107 (3)	0.105 (3)	0.002 (3)	−0.009 (3)	−0.062 (3)
O2	0.155 (3)	0.180 (4)	0.0575 (16)	−0.041 (3)	0.0267 (18)	−0.043 (2)
O3	0.0214 (7)	0.0398 (9)	0.0495 (9)	0.0015 (7)	0.0012 (6)	0.0023 (8)
O4	0.0373 (9)	0.0318 (9)	0.0742 (12)	0.0033 (8)	−0.0014 (8)	0.0039 (9)
O5	0.0272 (8)	0.0707 (13)	0.0439 (10)	0.0003 (9)	0.0073 (7)	−0.0007 (10)
O6	0.0444 (10)	0.0550 (12)	0.0641 (12)	0.0190 (10)	0.0003 (9)	−0.0045 (10)
O7	0.0323 (8)	0.0407 (9)	0.0507 (10)	−0.0021 (8)	0.0070 (7)	0.0013 (9)

### Geometric parameters (Å, °)

**Table:**

C1—C2	1.368 (5)	C11—C12	1.532 (4)
C1—C6	1.376 (5)	C11—H11	0.9800
C1—N1	1.473 (4)	C12—C13	1.528 (4)
C2—C3	1.376 (4)	C12—H12A	0.9700
C2—H2	0.9300	C12—H12B	0.9700
C3—C4	1.401 (4)	C13—C15	1.494 (6)
C3—H10	0.9300	C13—C14	1.537 (6)
C4—C5	1.379 (3)	C13—H13	0.9800
C4—C7	1.515 (3)	C14—H14A	0.9600
C5—C6	1.388 (4)	C14—H14B	0.9600
C5—H5	0.9300	C14—H14C	0.9600
C6—H6	0.9300	C15—H15A	0.9600
C7—O3	1.424 (3)	C15—H15B	0.9600
C7—C8	1.531 (3)	C15—H15C	0.9600
C7—H7	0.9800	N1—O2	1.195 (6)
C8—N2	1.489 (3)	N1—O1	1.228 (7)
C8—C9	1.528 (3)	N2—H2B	0.87 (3)
C8—H8	0.9800	N2—H2C	0.99 (3)
C9—O4	1.231 (3)	N2—H2A	0.95 (3)
C9—N3	1.323 (3)	N3—H3A	0.8600
C10—O6	1.236 (3)	O3—H4	0.8200
C10—O5	1.267 (3)	O7—H7W	0.9174
C10—C11	1.538 (3)	O7—H8W	0.8845
C11—N3	1.461 (3)		
			
C2—C1—C6	122.5 (3)	C12—C11—H11	109.3
C2—C1—N1	118.8 (4)	C10—C11—H11	109.3
C6—C1—N1	118.6 (4)	C13—C12—C11	116.2 (2)
C1—C2—C3	119.0 (3)	C13—C12—H12A	108.2
C1—C2—H2	120.5	C11—C12—H12A	108.2
C3—C2—H2	120.5	C13—C12—H12B	108.2
C2—C3—C4	120.2 (3)	C11—C12—H12B	108.2
C2—C3—H10	119.9	H12A—C12—H12B	107.4
C4—C3—H10	119.9	C15—C13—C12	112.1 (4)
C5—C4—C3	119.3 (3)	C15—C13—C14	111.3 (3)
C5—C4—C7	120.7 (2)	C12—C13—C14	109.2 (3)
C3—C4—C7	120.0 (2)	C15—C13—H13	108.1
C4—C5—C6	120.8 (3)	C12—C13—H13	108.1
C4—C5—H5	119.6	C14—C13—H13	108.1
C6—C5—H5	119.6	C13—C14—H14A	109.5
C1—C6—C5	118.1 (3)	C13—C14—H14B	109.5
C1—C6—H6	120.9	H14A—C14—H14B	109.5
C5—C6—H6	120.9	C13—C14—H14C	109.5
O3—C7—C4	112.48 (18)	H14A—C14—H14C	109.5
O3—C7—C8	107.33 (17)	H14B—C14—H14C	109.5
C4—C7—C8	110.03 (18)	C13—C15—H15A	109.5
O3—C7—H7	109.0	C13—C15—H15B	109.5
C4—C7—H7	109.0	H15A—C15—H15B	109.5
C8—C7—H7	109.0	C13—C15—H15C	109.5
N2—C8—C9	109.07 (18)	H15A—C15—H15C	109.5
N2—C8—C7	110.07 (17)	H15B—C15—H15C	109.5
C9—C8—C7	109.85 (18)	O2—N1—O1	123.3 (4)
N2—C8—H8	109.3	O2—N1—C1	119.1 (5)
C9—C8—H8	109.3	O1—N1—C1	117.6 (4)
C7—C8—H8	109.3	C8—N2—H2B	114.5 (19)
O4—C9—N3	124.7 (2)	C8—N2—H2C	111.6 (18)
O4—C9—C8	119.3 (2)	H2B—N2—H2C	107 (3)
N3—C9—C8	116.0 (2)	C8—N2—H2A	107.1 (19)
O6—C10—O5	124.3 (2)	H2B—N2—H2A	104 (3)
O6—C10—C11	118.5 (2)	H2C—N2—H2A	112 (3)
O5—C10—C11	117.1 (2)	C9—N3—C11	123.3 (2)
N3—C11—C12	109.3 (2)	C9—N3—H3A	118.3
N3—C11—C10	111.00 (19)	C11—N3—H3A	118.3
C12—C11—C10	108.64 (19)	C7—O3—H4	109.5
N3—C11—H11	109.3	H7W—O7—H8W	118.1
			
C6—C1—C2—C3	0.5 (5)	C7—C8—C9—O4	61.9 (3)
N1—C1—C2—C3	179.3 (3)	N2—C8—C9—N3	122.4 (2)
C1—C2—C3—C4	−0.3 (4)	C7—C8—C9—N3	−116.9 (2)
C2—C3—C4—C5	0.2 (4)	O6—C10—C11—N3	−155.5 (2)
C2—C3—C4—C7	−178.6 (2)	O5—C10—C11—N3	26.6 (3)
C3—C4—C5—C6	−0.2 (4)	O6—C10—C11—C12	84.4 (3)
C7—C4—C5—C6	178.6 (2)	O5—C10—C11—C12	−93.6 (3)
C2—C1—C6—C5	−0.5 (5)	N3—C11—C12—C13	54.7 (3)
N1—C1—C6—C5	−179.3 (3)	C10—C11—C12—C13	175.9 (3)
C4—C5—C6—C1	0.4 (4)	C11—C12—C13—C15	64.0 (4)
C5—C4—C7—O3	119.8 (2)	C11—C12—C13—C14	−172.3 (3)
C3—C4—C7—O3	−61.3 (3)	C2—C1—N1—O2	−174.0 (4)
C5—C4—C7—C8	−120.6 (2)	C6—C1—N1—O2	4.9 (5)
C3—C4—C7—C8	58.2 (3)	C2—C1—N1—O1	4.6 (6)
O3—C7—C8—N2	−47.8 (2)	C6—C1—N1—O1	−176.5 (4)
C4—C7—C8—N2	−170.52 (19)	O4—C9—N3—C11	1.5 (3)
O3—C7—C8—C9	−167.95 (18)	C8—C9—N3—C11	−179.66 (19)
C4—C7—C8—C9	69.4 (2)	C12—C11—N3—C9	−156.1 (2)
N2—C8—C9—O4	−58.8 (3)	C10—C11—N3—C9	84.1 (3)

### Hydrogen-bond geometry (Å, °)

**Table:**

*D*—H···*A*	*D*—H	H···*A*	*D*···*A*	*D*—H···*A*
O7—H7W···O5	0.92	1.87	2.679 (2)	146
O7—H8W···O4^i^	0.88	1.96	2.734 (3)	146
N2—H2B···O3^ii^	0.87 (3)	1.99 (3)	2.854 (3)	172 (3)
O3—H4···O5^iii^	0.82	1.79	2.612 (2)	175
N3—H3A···O7^i^	0.86	2.08	2.914 (3)	163
N2—H2C···O6^i^	0.99 (3)	1.96 (3)	2.811 (3)	143 (3)
N2—H2A···O7	0.95 (3)	1.89 (3)	2.801 (2)	158 (3)

Symmetry codes: (i) −*x*+1, *y*+1/2, −*z*+1; (ii) −*x*, *y*−1/2, −*z*+1; (iii) *x*−1, *y*, *z*.
